# Open Science Practices in Systematic Reviews From 2014 to 2024: A Cohort Study of 300 Systematic Reviews

**DOI:** 10.1002/cesm.70096

**Published:** 2026-07-18

**Authors:** Kenneth Färnqvist, Patrik Karlsson, Vera Wachtmeister, Patrick Vallance, Emma Sinervo

**Affiliations:** ^1^ Molecular Medicine and Surgery Karolinska Institutet Stockholm Sweden; ^2^ Department of Neurobiology, Care Sciences, and Society Karolinska Institutet Stockholm Sweden; ^3^ Department of Clinical Neurosciences Karolinska Institutet Stockholm Sweden; ^4^ Department of Physiotherapy, School of Primary and Allied Healthcare Monash University Melbourne Victoria Australia; ^5^ Department of Women's and Children's Health Karolinska Institutet Stockholm Sweden; ^6^ Centre for Epidemiology and Community Medicine Region Stockholm Stockholm Sweden

**Keywords:** cohort study, meta‐research, research on research

## Abstract

**Objectives:**

Open science practices, including the preregistration of protocols and the sharing of data and code, are increasingly promoted to enhance transparency, accessibility and the reuse of research outputs. However, systematic reviews, which are essential for informing clinical guidance and policy, often provide limited access to the materials needed for independent verification and reuse. Our objective was to estimate the prevalence of key open science indicators in interventional systematic reviews and to assess temporal changes by comparing our 2024 cohort with previously published cohorts from 2014 to 2020.

**Design:**

Cross‐sectional meta‐research study.

**Data Sources:**

MEDLINE, Embase, and CINAHL were searched in June 2024 for English‐language records indexed between 1 January and 1 February 2024.

**Eligibility Criteria:**

Interventional systematic reviews including human participants, a clearly defined PICO question, a systematic search strategy, a risk‐of‐bias assessment, and at least one meta‐analysis.

**Methods:**

Following our publicly registered protocol and STROBE guidance, records were randomly sampled and screened in duplicate until 300 eligible reviews were included. Data extraction was conducted in duplicate. We assessed protocol registration and availability, data sharing, code sharing, and preprint dissemination. Preprints were identified through extensive manual searches across more than 40 preprint servers and through author contact. Temporal changes were examined by comparing our 2024 cohort with previously published cohorts from 2014 to 2020 using pairwise risk ratios (RRs) with predefined equivalence ranges.

**Results:**

Among the 300 included reviews, cancer was the most common health condition (17%), and pharmacological or biological interventions were the most common intervention type (35%). The median number of primary studies included in each systematic review was 14 (IQR 9–23). Most reviews (73%) were published in journals with data and/or code‐sharing policies. In the 2024 cohort, 70% of systematic reviews reported a registered protocol or protocol availability and 61% included a data availability statement. However, only 24% shared underlying data, and analytical code was shared in 1% of reviews. Preprints were rare, occurring in 2% of the reviews. Reporting of protocol registration or availability increased substantially over time (2024 vs. 2014: RR 2.03, 95% CI 1.55–2.65; 2024 vs. 2020: RR 1.67, 95% CI 1.43–1.95), as did data availability statements (2024 vs. 2020: RR 1.98, 95% CI 1.63–2.40). Data sharing showed a nonlinear pattern, decreasing from 2014 to 2020 (RR 0.22, 95% CI 0.13–0.36) and increasing from 2020 to 2024 (RR 3.71, 95% CI 2.30–6.00), but remained uncommon overall. Code sharing was rare and showed no detectable change (OR 1.00, 95% CI 0.15–6.51). In the 2024 cohort, 3% of protocols were registered after manuscript submission.

**Conclusions:**

Several transparency indicators became more common over time, although trends differed across indicators. Yet the sharing of reusable research materials remains uncommon. Reusable data, analytic code, and preprints are rare outside the context of pandemics. These findings highlight a persistent gap between declarative openness and operational accessibility, emphasizing the necessity for more robust, verifiable mechanisms from journals and funders to reduce data distance and support independent verification and reuse of systematic review evidence. This gap between declared openness and practical accessibility continues to limit independent verification and reuse of systematic review evidence.

## 1 Background

In recent years, the importance of open science and transparency practices has gained recognition within the research community [1, 2]. Key practices include the sharing of data and analytic code and the preregistration of study protocols. Guidelines and recommendations have been established to encourage researchers to adopt these practices [3, 4, 5], with the aim of improving the quality and transparency of research [6]. These practices are commonly considered key indicators of open science and research transparency [7]. The number of studies adopting these practices can be considered an indicator of the extent to which open science is adopted in research.

The replication of systematic reviews is particularly important due to their frequent use in informing policymaking, clinical decision‐making, and guidelines [8]. Replicating a study can enhance its trustworthiness; however, conflicting results or conclusions may undermine the credibility of the original study [9], a concern highlighted by the broader replication crisis in several scientific fields [10, 11]. Previous methodological research has demonstrated that various choices made in reviews can significantly affect the results and conclusions [12]. Thus, the intentional replication of systematic reviews is necessary to evaluate the reliability of previously generated knowledge [9]. Access to underlying data and analytic materials may facilitate the verification, reuse and updating of systematic reviews [13, 14]. The sharing of aggregated data has several benefits, including facilitating independent verification of findings, enabling additional analyses beyond those investigated in the current review, minimizing the risk of faulty data extraction, and facilitating the creation of umbrella reviews [13, 14]. Recent meta‐research has challenged the assumption that systematic reviews are inherently reproducible and replicable. Independent investigations have shown that repeating literature searches, study selection, data extraction, and meta‐analyses can yield discrepant results, often because review methods are incompletely reported and underlying review materials are limited in availability. These findings underscore the importance of protocol registration and the sharing of review data and analytic code as mechanisms for improving the transparency, reproducibility, and replicability of systematic reviews [15, 16].

Despite increasing attention to open science, data sharing in systematic reviews remains rare. In a cross‐sectional sample of 300 systematic reviews published in 2020, only 7% provided access to the underlying data or analytical code [17]. Similarly, only 31% of the articles included a data availability statement. Of those, most statements either offered access “on request” or implied that sharing was unnecessary [18]. Furthermore, although authors indicate that data are “available upon request,” actual compliance is very low. In a large survey of 3416 manuscripts with data availability statements, fewer than 7% of authors who promised to share data upon request actually provided it [19]. These findings suggest a substantial gap between declared openness and the actual accessibility of review materials.

Recent large‐scale reproducibility research in primary studies further underscores the importance of access to both data and analytic code [2]. In a stratified sample of 600 papers, only 20% provided both data and code. Among these, approximately 91% of findings were reproducible. In contrast, reproducibility declined to 73% when only data were available and to 38% when researchers had to reconstruct datasets and analytical workflows from source data. These findings suggest that access to underlying materials is a key determinant of whether published results can be independently verified, reinforcing the need to assess not only reported transparency but also the availability of reusable research outputs.

**Figure 1:**
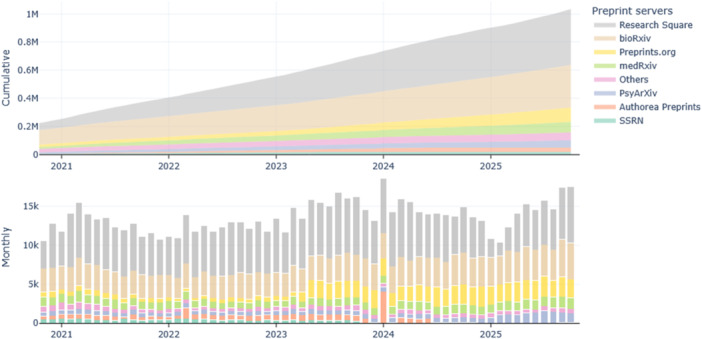
The figure illustrates the growth of preprints indexed in Europe PMC over time. The upper panel shows the cumulative number of indexed preprints by server, demonstrating a steady and accelerating increase across several platforms. The lower panel presents monthly indexing counts, highlighting sustained contributions from several major preprint servers and reflecting the growing role of preprints in scientific communication in recent years. Europe PMC. *Preprints in Europe PMC*. Available from: https://europepmc.org/Preprints. Accessed 12 Jan 2026.

Another critical aspect of open science is publishing research findings as preprints, which has increased in recent years (Figure 1). Preprints allow research articles to be freely accessible to large audiences prior to journal publication. However, it is important to note that preprints do not always undergo external quality control measures [20]. Publishing articles in the preprint format offers several advantages. The conventional process of submitting a manuscript to a journal can take several months or longer before publication. In contrast, preprints allow research findings to be disseminated immediately and enable rapid feedback from the scientific community. Feedback may also come from a broader audience rather than only a small number of peer reviewers, and preprints are freely accessible, allowing research to reach a wider readership. Furthermore, no rigid structural requirements tied to a specific journal need to be met, meaning that the content itself becomes the primary focus. Articles that were previously available as preprints have also been shown to receive higher Altmetric scores and more citations [20, 21, 22]. Preprints may also help reduce the risk of publication bias by allowing research findings to be disseminated even if they are not subsequently published in peer‐reviewed journals [23]. They may further enhance transparency by allowing readers to track how a manuscript evolves from its initial version to the final peer‐reviewed publication [24]. Despite this, it is essential to acknowledge that preprints have some limitations [20]. One apparent pitfall is the lack of quality control prior to dissemination. This could potentially result in alterations to clinical treatment and escalate health‐related anxiety among individuals [25]. Hence, it is crucial to meticulously scrutinize preprints and their sources before relying on them for decision‐making [20].

To the best of our knowledge, no study with a broader scope (i.e., not restricted to COVID‐19) has systematically assessed the number of systematic reviews initially published as preprints. In a cohort of COVID‐19 prevention reviews registered in PROSPERO, 13 of 47 (28%) were eventually published, including 6 as preprints, 6 as journal articles, and 1 as both [26]. Another investigation of COVID‐19 treatment reviews also highlighted the substantial use of preprints but reported variable uptake across topics [27]. More recently, a cross‐sectional study of 246 COVID‐19 systematic reviews found that 14% had been published as preprints, with similar proportions reported in other reviews from the same period [28]. Collectively, these findings suggest that while preprints are used for systematic reviews, their uptake has been limited and context‐dependent, and little is known outside the COVID‐19 field. More broadly, studies across scientific fields suggest that approximately 65%–75% of preprints are eventually published in peer‐reviewed journals [22]. In orthopedics, for instance, a corresponding journal publication was identified for 52% (762 of 1471) of the preprints [29].

### 1.1 Problem Statement

Although evidence on open science practices in original research [30, 31, 32], only a limited number of studies have assessed these practices in systematic reviews. Existing studies have largely focused on reporting quality, search reproducibility, and transparency in specific disciplines, such as pediatric surgery, otorhinolaryngology, oncology, and psychology [33, 34, 35, 36, 37, 38]. These studies consistently show important gaps in transparency, reporting, and data‐sharing practices.

Evidence regarding preprints in systematic reviews is scarce. To date, published investigations have been conducted almost exclusively in the context of COVID‐19. These studies suggest that although systematic reviews are occasionally disseminated as preprints, their uptake is limited, highly context‐dependent, and typically investigated only as a secondary outcome [26, 27, 28].

### 1.2 Aim and Research Questions

To our knowledge, no research outside the context of COVID‐19 has systematically investigated the prevalence of systematic reviews disseminated as preprints before journal publication. Furthermore, existing evidence is available but largely reflects earlier publication periods and may not represent current practice. Consequently, the primary objective of this study was to evaluate the prevalence of key open‐science practices in contemporary systematic reviews. We selected protocol registration, protocol availability, data sharing, code sharing, and preprint dissemination because they represent widely recognized and measurable indicators of open science. Together, these practices increase the transparency, accessibility, and reusability of research and have been used in previous meta‐research studies evaluating open science practices in systematic reviews. Specifically, we aimed to:

- Estimate the frequency of protocol registration, protocol availability, data sharing, and code sharing in a contemporary cohort of systematic reviews.
- Estimate the proportion of systematic reviews that were disseminated as preprints before publication in a peer‐reviewed journal.
- Compare these indicators across publication periods (2014, 2020, and 2024) to assess temporal changes in open science practices, using previously published data for the 2014 and 2020 cohorts and newly collected data for the 2024 cohort [17, 18].

## 2 Methods

This cross‐sectional cohort study followed the Strengthening the Reporting of Observational Studies in Epidemiology (STROBE) guidelines. Before the search commenced, a public study protocol (https://osf.io/rdpfa) was established.

### 2.1 Deviations From Protocol

Per protocol, we planned to (i) report the percentage of reviews meeting each predefined open‐science indicator and the frequency of preprints, (ii) present quantitative results as absolute numbers, proportions, or medians, and (iii) conduct an inductive qualitative analysis following prior categorizations. In addition, prior to data analysis, we specified between‐year comparisons to enhance interpretability and enable direct comparability with prior meta‐research (2014 and 2020 cohorts) [17, 18]; see Statistical analysis for details. No other changes were made to the outcomes, eligibility criteria, or data items.

### 2.2 Searches for Studies

The full search strategies for all databases are available in the Material S1.

A literature search was performed using the MEDLINE, Embase, and CINAHL databases. The search was conducted in June 2024.

The search strategy was developed in MEDLINE (Ovid) in collaboration with librarians at the Karolinska Institutet University Library. Medical Subject Headings (MeSH) and free‐text terms were identified for each search concept. The search was then translated in part into other databases using the Polyglot Search Translator [39]. Databases were searched from January 1 to February 1, 2024. English language restrictions were applied due to time and resource constraints. The strategies were peer‐reviewed by another librarian before their execution. Deduplication was performed in Covidence (Veritas Health Innovation, Melbourne, Australia).

### 2.3 Sampling

We gathered systematic reviews from all databases in accordance with the power calculations mentioned in a previous publication [19]. After collecting and deduplicating the reviews, a random sample of the first 2000 unique records was selected using Microsoft Excel. Subsequently, two researchers (KF and ES) independently screened the titles and abstracts of the remaining records against the eligibility criteria using Covidence. Subsequently, the full texts of potentially eligible records were independently assessed by the same 2 researchers (KF and ES) in a randomized order against the eligibility criteria until the desired sample size of 300 systematic reviews was achieved. Any discrepancies during each screening stage were resolved through discussion or consultation with a third author (VW).

### 2.4 Eligibility Criteria

A systematic review was considered eligible if it had a distinct PICO (population, intervention, comparator, outcome) question, included human participants, used a systematic search strategy to identify relevant literature, assessed the risk of bias in the included studies, and included at least one meta‐analysis (including both network and pairwise meta‐analyses).

We excluded reviews without included studies, review protocols, qualitative evidence syntheses, rapid reviews, scoping reviews, review overviews, systematic reviews of diagnostic accuracy, prognostic reviews, and methodological reviews.

### 2.5 Outcomes and Data Collection

The temporal comparisons were based on previously published cohorts from 2014 to 2020 that used broadly comparable designs, including random sampling of systematic reviews of interventions, duplicate screening and data extraction procedures, and target sample sizes of 300 reviews [17, 18]. Although minor methodological differences between cohorts may exist, the studies were considered sufficiently comparable to support descriptive assessments of temporal trends. Data for the 2024 cohort were extracted as described below.

Data extraction was performed in duplicate. KF extracted data from all 300 reviews, whereas PK, PV, and VW independently extracted data from a random subset of 100 reviews. Any discrepancies were resolved through discussion with a third author (ES). We collected data using a customized and pretested data extraction sheet in Microsoft Excel. The categories for data extraction included general information about the review, such as the title, topic, publication date, type of intervention, health condition investigated, and number of included studies. We also collected information on the outcomes of interest in this study (data sharing, code sharing, pre‐registration, protocol availability, and preprints), including the frequency of each outcome and the number of studies included. If we were unable to obtain particular information on an outcome, we first examined the materials available on the journal website. Next, we emailed the corresponding authors for the missing data.

Preprints were identified through manual searches across preprint servers. First, we searched OSF Preprints using the title and author names. Subsequently, we searched specific preprint servers using a predefined list of repositories (updated February 2022) from the Kirkham et al. study [40]. Third, we searched CrossRef for corresponding DOIs. Finally, we attempted to contact the corresponding authors directly to obtain missing information or identify potential preprints (see the email template in the Material S1).

### 2.6 Data Analysis

We summarized the characteristics using counts/percentages and medians with interquartile ranges. For each indicator, we estimated pairwise risk ratios (RRs) with Wald 95% confidence intervals, comparing 2024 versus 2014 and 2024 versus 2020. When events were rare (numerator <5 or prevalence <5% in either group), we used penalized likelihood logistic regression to obtain odds ratios, interpreted as approximations to RRs when events were rare in both groups. To aid interpretation, we predefined an equivalence range of RR 0.90–1.10 to distinguish practically important differences from trivial differences. This threshold was adopted from the 2020 comparator study [17] to enable direct comparison across cohorts and was specified a priori. Consistent with the previous study, differences exceeding 10% in either direction were considered potentially important, whereas smaller differences were considered unlikely to be practically meaningful. Analyses were conducted in R (e.g., *epitools* for RRs and Wald confidence intervals [CIs]; *logistf* for penalized models).

## 3 Results

### 3.1 Search Strategy

**Figure 2:**
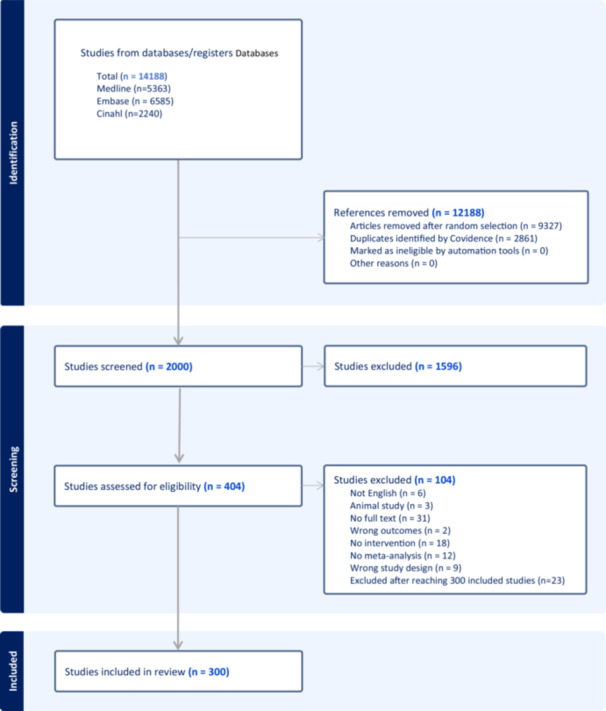
Flowchart illustrating the identification, screening, eligibility assessment, and inclusion of studies in the study, including reasons for exclusion at the full‐text stage.

Our search identified 14,188 records. After removing duplicates (*n* = 2861), 11,327 records remained. From these, a random sample of 2000 records was selected for title and abstract screening. Of these, 404 articles were considered potentially eligible and retrieved for full‐text assessment. We continued full‐text screening until our predefined target sample of 300 eligible systematic reviews was reached. As such, some of the retrieved reports were not assessed further once the target sample size was reached (Figure 2).

### 3.2 Characteristics of the 2024 Cohort

Table 1 presents the characteristics of the included systematic reviews (the full dataset is presented in the Material S1). Among the included systematic reviews, cancer was the most frequently studied health condition (17%, 51/300), followed by cardiovascular disease (13.3%), neurological conditions (11.3%, 34/300), and mental health conditions (9%, 27/300). Reviews addressing musculoskeletal (6%, 18/300) and infectious diseases (5%, 15/300) were less common, whereas a substantial proportion focused on other health conditions (38.3%, 115/300).

**Table 1:** Cohort characteristics.

Section	Characteristic	Value
Health condition	Cancer	51 (17%)
	Cardiovascular	40 (13.3%)
	Infectious disease	15 (5%)
	Mental health	27 (9%)
	Musculoskeletal	18 (6%)
	Neurological	34 (11.3%)
	Other	115 (38.3%)
Intervention	Digital/Educational/Care‐delivery	15 (5%)
	Exercise/Physical/Rehabilitation	31 (10.3%)
	Other/Complementary	61 (20.3%)
	Pharmacological/Biological	105 (35.0%)
	Psychological/Behavioral	23 (8%)
	Surgical/Procedural/Invasive	65 (22%)
Methods	Included studies per review	Median 14 (IQR 9‐23)
	Risk of bias tool: Other	95 (26.3%)
	Risk of bias tool: Jadad scale	12 (3.3%)
	Risk of bias tool: ROBINS‐I	29 (8%)
	Risk of bias tool: RoB 1	108 (30%)
	Risk of bias tool: RoB 2	117 (32.4%)
Journal policy	Data/code sharing policy: No	82 (27%)
	Data/code sharing policy: Yes	218 (73%)
	Policy expectation: Encouraged	138 (46%)
	Policy expectation: Expected	74 (25%)
	Policy expectation: Mandatory	6 (2%)
	Policy expectation: No policy	82 (27%)

Abbreviation: IQR, interquartile range.

Pharmacological or biological interventions were the most common intervention category (35.0%), followed by surgical, procedural, or invasive interventions (22%) and other or complementary interventions (20.3%). Exercise, physical, or rehabilitation interventions accounted for 10.3% of the reviews, psychological or behavioral interventions for 8%, and digital, educational, or care delivery interventions for 5%.

The median number of primary studies included in each review was 14 (interquartile range [IQR], 9–23). The most commonly used risk of bias (RoB) tools were RoB‐2 (32.4%) and RoB‐1 (30%), followed by other tools (26.3%). ROBINS‐I was used in 8% (29/300) of the reviews and the Jadad scale was rarely applied (3.3%).

Most reviews were published in journals with data and/or code‐sharing policies (73%). Among journals with a policy, data or code sharing was most commonly encouraged (46%), followed by expected (25%) and mandatory (2%) policies.

### 3.3 Open Science Indicators in the Contemporary Cohort

Table 2 summarizes the prevalence of key open science indicators in our 2024 cohort of systematic reviews. Among the included reviews, 210 of 300 (70%) reported a registered protocol or protocol availability. Most protocols were registered before manuscript submission (187/300, 62%), whereas a small proportion were registered after submission (10/300, 3%); no protocols were registered for the remaining reviews (103/300, 34%).

**Table 2:** Open science indicators across years.

Open science indicator	2014	2020	2024
Registered protocol (%)	38/110 (35%)	123/294 (42%)	210/300 (70%)
Protocol before submission (%)	–	–	187/300 (62%)
Protocol after submission	–	–	10/300 (3%)
Data availability statement (%)	–	93/300 (31%)	183/300 (61%)
Any data shared (%)	33/110 (30%)	19/294 (6%)	72/300 (24%)
Code shared (%)	–	2/300 (1%)	2/300 (1%)
Preprints (%)	–	–	3/300 (2%)

Data availability statements were reported in 183 of the 300 reviews (61%). However, the actual sharing of reusable data was less common, with 72 reviews (24%) providing access to the extracted data or datasets underlying the analyses.

Code sharing was rare. Only 2 of 300 reviews (1%) shared the analytical code used for the meta‐analyses.

### 3.4 Preprint Dissemination

Preprints were rare among the included systematic reviews. In the 2024 cohort, only 3 of 300 reviews (2%) had been disseminated as preprints before publication in a peer‐reviewed journal.

### 3.5 Temporal Changes in Open Science Practices

**Figure 3:**
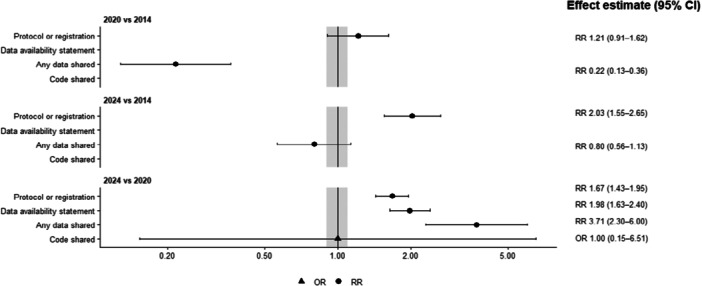
Temporal changes in open science practices in systematic reviews (2014–2024). Points show effect estimates comparing the 2024 cohort with previously published cohorts from 2014 to 2020 (risk ratios [RR]; odds ratios [OR] for rare events) with 95% confidence intervals. The vertical line indicates no difference (RR = 1.0). The shaded band indicates the predefined equivalence range (RR 0.90–1.10), used to distinguish meaningful change from negligible differences. Numerical estimates are presented in the right‐hand column.

We observed distinct temporal changes when comparing our 2024 cohort with earlier cohorts from 2014 to 2020 (Figure 3, Table 2).

The protocol or registration increased consistently over time. Compared with that in 2014, the likelihood of reporting a protocol or registration was higher in 2020 (RR 1.21, 95% CI 0.91–1.62) and substantially higher in 2024 (RR 2.03, 95% CI 1.55–2.65). A further increase was observed between 2020 and 2024 (RR 1.67 [95% CI 1.43–1.95]). All point estimates were outside the predefined equivalence range (RR 0.90–1.10), indicating practically important differences.

Data availability statements showed a marked increase between 2020 and 2024 (RR 1.98, 95% CI 1.63–2.40), exceeding the equivalence range and suggesting a substantial improvement in reporting over this period.

In contrast, data sharing displayed a nonlinear pattern. Reporting decreased from 2014 to 2020 (RR 0.22, 95% CI 0.13–0.36), followed by no clear difference between 2014 and 2024 (RR 0.80, 95% CI 0.56–1.13); the latter estimate overlapped both the null and equivalence ranges. However, a pronounced increase was observed from 2020 to 2024 (RR 3.71, 95% CI 2.30–6.00), indicating a substantial relative change in the most recent period.

Code sharing remained rare between 2020 and 2024. In the comparison between 2020 and 2024, estimates were derived using penalized likelihood logistic regression due to sparse data, yielding an odds ratio of 1.00 (95% CI 0.15–6.51), consistent with no detectable difference.

## 4 Discussion

### 4.1 Principal Findings and Interpretation

Recent studies have directly evaluated the reproducibility and replicability of systematic reviews. In contrast, the present study focused on open science practices that may facilitate transparency, verification, reuse, and future reproducibility efforts. In this cross‐sectional cohort of systematic reviews, several open science indicators were frequently reported, but the sharing of reusable research materials remained uncommon. In the 2024 cohort, 70% of reviews reported a registered protocol and 61% included a data availability statement. However, only 24% shared underlying data, and only 1% shared analytical code. When we contacted the authors to request additional materials, only 39 of 300 responded.

We also observed differing temporal patterns across indicators. Protocol registration and reporting of data availability statements became more common over time, whereas data sharing declined between 2014 and 2020 before partially recovering in 2024. The apparent decline in data sharing between 2014 and 2020 may partly reflect the substantial decrease in the proportion of Cochrane reviews over time, as Cochrane reviews have historically been subject to more stringent requirements for sharing review materials. Nevertheless, these changes have not consistently translated into greater sharing of reusable data or analytical code, a pattern also observed in prior meta‐research [17, 18].

Collectively, these findings suggest an increasing emphasis on declarative transparency, whereas the sharing of reusable research materials remains limited, echoing concerns raised by Page et al. [18] and Gabelica et al. [19]. Systematic reviews are increasingly signaling openness through complying with registration and data availability statements but frequently fail to provide the materials necessary to independently, verify, scrutinize or extend their findings.

### 4.2 Comparison with Previous Research

Our findings are consistent with those of prior meta‐research, demonstrating that the implementation of open science practices in systematic reviews remains limited. Nguyen et al. [17] identified that only 7% of systematic reviews shared any data or code. Similarly, Page et al. [18] reported that only 31% of articles included a data availability statement. Gabelica et al. [19] also demonstrated that fewer than 7% of authors complied with data‐sharing requests when data were stated to be “available upon request.” Discipline‐specific investigations have further highlighted these problems. Research in pediatric surgery [33], otorhinolaryngology [34], oncology [35], psychiatry [36], psychology [38], and psychotherapy [37] has highlighted significant deficiencies in transparencyand data sharing. Furthermore, Koffel and Rethlefsen [41] demonstrated poor reproducibility of search strategies in systematic reviews published in high‐impact journals. These findings suggest that systematic reviews have consistently failed to meet best open science practices across various disciplines.

### 4.3 Preprints, Protocol Registration, and Timing

To date, the incorporation of preprints into systematic reviews has been predominantly examined within the context of the COVID‐19 pandemic. Research on COVID‐19 prevention and treatment reviews has indicated a variable integration of preprints, frequently as a secondary outcome [26, 27]. A more extensive cross‐sectional analysis revealed that approximately 14% of COVID‐19 systematic reviews were initially disseminated as preprints [28]. Our findings contribute to this body of literature by illustrating that systematic reviews are infrequently disseminated as preprints outside the pandemic context. Although preprints may accelerate dissemination, transparency and reuse ultimately depend on access to the underlying review materials, irrespective of publication format. In our cohort of systematic reviews, only 24% shared underlying data and just 1% provided analytical code, limiting opportunities for independent verification and reuse. These findings highlight the importance of ensuring that transparency extends beyond publication and includes access to reusable research materials [17, 18]. Although most registered protocols were registered before manuscript submission, a small proportion were registered afterwards. Protocol registration after study initiation or manuscript submission is unlikely to fulfill the primary purpose of prospective registration, namely, reducing selective reporting and increasing transparency regarding planned methods. Nevertheless, distinguishing between prospective and retrospective registration may provide a more nuanced picture of registration practices than reporting registration alone.

### 4.4 Strengths and Limitations

The principal strengths of this study include its adherence to a registered protocol, compliance with the STROBE guidelines, and incorporation of a large, contemporary cohort of systematic reviews. We extend previous research by systematically searching over 40 biomedical preprint servers and directly contacting authors, thereby providing a more accurate estimate of accessible review materials than previous studies that relied solely on published statements [17, 18]. Despite this, our study has several limitations. Our sample was confined to interventional systematic reviews indexed in selected medical databases during a specific timeframe, which may have impacted generalizability. Identifying preprints and web‐based materials is inherently challenging [33, 41], and some preprints may have been overlooked despite extensive searches. In addition, the temporal comparisons relied on cohorts derived from separate meta‐research studies. Although the cohorts used broadly comparable designs and eligibility criteria, minor methodological differences may have influenced the observed temporal trends. These comparisons should therefore be interpreted with caution. Finally, contacting authors may introduce response bias, as researchers with more robust transparency practices might be more inclined to respond; this limitation has also been noted in previous studies [19].

### 4.5 Policy and Conceptual Implications

Our findings are consistent with recent theoretical advances in open science that emphasize the concept of “data distance,” defined as the gap between data producers and potential reusers [42, 43, 44]. In our study, attempts to obtain additional materials through author contact yielded limited results; most authors did not respond (87%, only 39/300 (13%) responded), and no additional materials were made available beyond those already openly shared. This pattern illustrates that, in practice, review materials are often difficult to access, even when authors are identifiable, and that “available on request” statements rarely translate into actual data provision.

### 4.6 Implications for Research and Practice

Our findings underscore the need for more stringent journal and funder policies to effectively advance data and code sharing in systematic reviews. Reliance on “available on request” statements is insufficient, as evidenced by consistently poor compliance [19]. The implementation of established frameworks, such as the Transparency and Openness Promotion Guidelines, data citation principles, and repository standards with persistent identifiers, could facilitate the transition from nominal transparency to meaningful transparency and reuse in this field [45]. Future meta‐research should evaluate the accountability of access claims more systematically, including fulfillment rates and response times. The development and testing of a quantitative “data distance index” may further operationalize openness in evidence synthesis research.

## 5 Conclusions

This study demonstrates that temporal trends differed across transparency indicators. While protocol registration and data availability statements became more common, substantial deficiencies remain in the sharing of reusable data and analytical code. While the dissemination of systematic reviews through preprints is possible, it remains infrequent outside of the pandemic context and does not ensure transparency, verification, or reuse. By incorporating temporal trend analyses, comprehensive searches across more than 40 preprint servers, and direct author contact, this study provides a more comprehensive assessment of the accessibility of systematic review evidence.

## Author Contributions

**Kenneth Färnqvist:** conceptualization, methodology, formal analysis, investigation, data curation, writing – original draft, writing – review and editing, project administration, funding acquisition. **Patrik Karlsson:** conceptualization, investigation, writing – review and editing. **Vera Wachtmeister:** conceptualization, investigation, writing – review and editing. **Patrick Vallance:** conceptualization, investigation, writing – review and editing. **Emma Sinervo:** conceptualization, methodology, formal analysis, investigation, writing – review and editing, funding acquisition. All authors reviewed the results and approved the final manuscript.

## Ethics Statement

This study did not require ethical approval because it was based exclusively on the analyses of publicly available published data and did not involve the collection or analysis of individual‐level personal data. No interaction with human participants occurred, and no identifiable or sensitive information was accessed. In accordance with applicable national regulations, research based solely on the secondary use of published literature does not require review by a research ethics committee.

## Conflicts of Interest

All authors have approved the final version of the manuscript and declare no conflicts of interest.

## PPI

Patients and members of the public were not involved in the design, conduct, reporting, or dissemination plans of this meta‐research study.

## Transparency Statement

The guarantor confirms that this manuscript is an honest, accurate, and transparent report of the study; that no significant aspects of the study have been omitted; and that any differences from the original study plan have been clarified.

## Supporting information


Supporting File 1

Supporting File 2

Supporting File 3

## Data Availability

All data extracted and analyzed during this study, together with the study protocol, data extraction forms, and analysis code, are publicly available via the Open Science Framework (OSF) at: https://osf.io/rdpfa. The dataset consists exclusively of information derived from published systematic reviews and does not contain individual‐level or sensitive data. A preprint of this manuscript is also available on https://osf.io/preprints/metaarxiv/x59m3_v1.
