# Supplementary material for: Open Science Practices in Systematic Reviews From 2014 to 2024: A Cohort Study of 300 Systematic Reviews
Source: Cochrane Evid Synth Methods. 2026 Jul 18;4(4):e70096. doi: 10.1002/cesm.70096 (PMC13387410; doi:10.1002/cesm.70096)
Supplement: Supplementary file 1 — Supporting File 1 [file CESM-4-e70096-s003.docx]

Documentation of search strategies

University Library search consultation group

Date: June 2024

Topic/research question: Systematic reviews from jan 2024

Name of researcher(s): Kenneth Färnqvist,

Librarian(s): Emma-Lotta Säätelä & Narcisa Hannerz.

Databases:

1. Medline (Ovid)
2. Embase (embase.com)
3. Cinahl (Ebsco)

Total number of hits:

- Before deduplication: 14,188
- After deduplication: 11,327

Search strategy

A literature search for systematic reviews published in January 2024 was performed in the following databases: Medline (Ovid), Embase (embase.com) and Cinahl (Ebsco). The last search was conducted 2024-06-11.

1. Medline

Reports not retrieved

(n = )

| Interface: Ovid MEDLINE(R) ALL  Date of Search: 11 June 2024  Number of hits: 5,363  Comment: In Ovid, two or more words are automatically searched as phrases; i.e. no quotation marks are needed | Field labels   - .ti,kf. = title, abstract and author keywords - adjx = within x words, regardless of order - * = truncation of word for alternate endings - ep=electronic publication date - dt=publication dae - pt=publication type |
| --- | --- |
| Database(s): **Ovid MEDLINE(R) ALL** 1946 to May 23, 2024 Search Strategy:   \| **#** \| **Searches** \| **Results** \| \| --- \| --- \| --- \| \| 1 \| meta analysis.pt. \| 201016 \| \| 2 \| meta analy*.ti,kf. \| 213784 \| \| 3 \| systematic review.pt. \| 261397 \| \| 4 \| (systematic adj3 review).ti,kf. \| 266843 \| \| 5 \| or/1-4 \| 440923 \| \| 6 \| 202401*.ep,dt. \| 166309 \| \| 7 \| 5 and 6 \| 5363 \| | |

2. Embase

| Interface: embase.com  Date of Search: 11 June 2024  Number of hits: 6,585  Comment: Emtree is the controlled vocabulary in Embase | Field labels   - /exp = exploded Emtree term - /de = non exploded Emtree term - ti,kw = title, abstract and author keywords - NEAR/x = within x words, regardless of order - * = truncation of word for alternate endings - pd=source publication date |
| --- | --- |
| \| No. \| Query \| Results \| \| --- \| --- \| --- \| \| #9 \| #6 NOT #7 AND ([article]/lim OR [review]/lim) \| 6585 \| \| #8 \| #6 NOT #7 \| 7369 \| \| #7 \| #6 AND [article in press]/lim \| 4096 \| \| #6 \| (#1 OR #2 OR #3 OR #4) AND ('2024-01-01':pd OR '2024-01-02':pd OR '2024-01-03':pd OR '2024-01-04':pd OR '2024-01-05':pd OR '2024-01-06':pd OR '2024-01-07':pd OR '2024-01-08':pd OR '2024-01-09':pd OR '2024-01-10':pd OR '2024-01-11':pd OR '2024-01-12':pd OR '2024-01-13':pd OR '2024-01-14':pd OR '2024-01-15':pd OR '2024-01-16':pd OR '2024-01-17':pd OR '2024-01-18':pd OR '2024-01-19':pd OR '2024-01-20':pd OR '2024-01-21':pd OR '2024-01-22':pd OR '2024-01-23':pd OR '2024-01-24':pd OR '2024-01-25':pd OR '2024-01-26':pd OR '2024-01-27':pd OR '2024-01-28':pd OR '2024-01-29':pd OR '2024-01-30':pd OR '2024-01-31':pd) \| 11465 \| \| #5 \| #1 OR #2 OR #3 OR #4 \| 676578 \| \| #4 \| (systematic NEAR/3 review):ti,kw \| 312243 \| \| #3 \| 'systematic review'/de \| 469006 \| \| #2 \| 'meta analy*':ti,kw \| 260630 \| \| #1 \| 'meta analysis'/exp \| 316771 \| | |

3. Cinahl

| Interface: Ebsco  Date of Search: 11 June 2024  Number of hits: 2,240 | Field labels   - TI = title - AB = abstract - Nx = within x words, regardless of order - * = truncation of word for alternate endings - PT=publication type   Note: sometimes “quotation marks” are needed for single search terms to avoid automatic term mapping (lemmatization) |
| --- | --- |
| \| **#** \| **Query** \| **Results** \| \| --- \| --- \| --- \| \| S5 \| S1 OR S2 OR S3 OR S4  Limiters - Publication Date: 20240101-20240131 \| 2,240 \| \| S4 \| TI systematic N3 review OR AB systematic N3 review \| 162,098 \| \| S3 \| PT systematic review \| 159,693 \| \| S2 \| TI "meta analy*" OR AB "meta analy*" \| 116,454 \| \| S1 \| PT meta analysis \| 57,957 \| | |
